# Supplementary material for: Acute psychological stress does not influence joint position reproduction performance in the elbow joint
Source: PLoS One. 2025 Mar 3;20(3):e0319061. doi: 10.1371/journal.pone.0319061 (PMC11875289; doi:10.1371/journal.pone.0319061)
Supplement: Attachment — Submitted filename: Response to Reviewers.docx [file pone.0319061.s001.docx]

Dear Dr. Koncz,

Thank you for submitting your manuscript to PLOS ONE. After careful consideration, we feel that it has merit but does not fully meet PLOS ONE’s publication criteria as it currently stands. Therefore, we invite you to submit a revised version of the manuscript that addresses the points raised during the review process.

**ACADEMIC EDITOR:**

Dear Authors
The manuscript underwent evaluation by two expert reviewers and an academic editor. Both reviewers concluded that the manuscript is well-written and addresses a significant research area. I believe that the manuscript will be significantly improved after revision.

Dear Editor

Thank you for the positive feedback. We addressed all suggestions and remarks; our responses to the comments can be found below.

Comments:
1. Authors should address the limitations of the sample, which consists only of young university students.

Thank you for this comment, the limitation section is extended by the following sentence: An important limitation of the current study is that the sample was homogeneous as the study involved first year university students, 86% of whom were female. This limits the generalizability of the findings. For future studies, it is recommended to use a more diverse, preferably representative sample. It is possible that the stress effect on proprioceptive accuracy is not linear and shows high variability across individuals. Future studies should also test these possibilities.

2. Numerous repetitions are evident, especially in the discussion section.

Thank you. The authors have tried to eliminate repetitions in the manuscript

3. Remove citations from the abstract.

We have removed the references from the abstract as suggested

4. Authors should explain their rationale for selecting only heart rate (HR), high frequency (HF) of heart rate variability (HRV), and root mean square of successive differences (RMSSD) from ECG data.

Thank you for this suggestion, the rationale for selecting these variables can now be found in the measurements section, please see the following sentences: “Under resting conditions, HR is controlled almost exclusively by the parasympathetic nervous system [36]; thus, an increase in HR in this domain indicates decreasing parasympathetic (vagal) activity. Similarly, as certain indices of HRV, such as the high-frequency domain of HRV (HF) and the root mean square of successive differences between normal heartbeats (RMSSD), correlate well with parasympathetic impact on the heart [36,37], decreasing values indicate decreasing parasympathetic activation.”

5. In electrodermal activity, skin conductance response (SCR) and skin conductance level (SCL) are frequently utilized. Nonetheless, the present study exclusively employed SCL. Kindly provide a rationale.

As we were curious about tonic changes of the sympathetic activation and not about acute responses to individual stimuli, SCL was the obvious choice. This is explained in the revised version.

6. Authors must present a comprehensive methodology for the preprocessing of ECG and EDA data.

Data processing is explained in the measurement section. For SCL: “Possible artifacts were visually checked for all participants. “ and “After excluding the artifacts, the BioTrace+ software was used to calculate mean SCL for the first 60 sec (pre) and final 60 sec period (post) of the quiz”.
In case of ECG please see the following sentence: “ ECG data were analysed with the KubiosHRV Premium 3.5.0. software (Kubios Oy, Finland). The software automatically detects QRS-complexes, identifies and corrects for ectopic and missing heartbeats, and finally calculates the required indices (Lipponen & Tarvainen, 2019; Tarvainen et al., 2014). RR interval detrending was carried out using the smoothn priors method with a smoothing parameter of 500. The level of automatic quality detection was set to medium, beat correction was automatic with an acceptance threshold of 5%.”

In the statistical analysis section, we mention that “SCL, HF, and RMSSD data were log-transformed to reduce deviation from normal distribution”

Please revise your manuscript based on my comments and other comments given by both the reviewers.

Please submit your revised manuscript by Jan 31 2025 11:59PM. If you will need more time than this to complete your revisions, please reply to this message or contact the journal office at [plosone@plos.org](mailto:plosone@plos.org). When you're ready to submit your revision, log on to https://www.editorialmanager.com/pone/ and select the 'Submissions Needing Revision' folder to locate your manuscript file.

Please include the following items when submitting your revised manuscript:

- A rebuttal letter that responds to each point raised by the academic editor and reviewer(s). You should upload this letter as a separate file labeled 'Response to Reviewers'.
- A marked-up copy of your manuscript that highlights changes made to the original version. You should upload this as a separate file labeled 'Revised Manuscript with Track Changes'.
- An unmarked version of your revised paper without tracked changes. You should upload this as a separate file labeled 'Manuscript'.

If you would like to make changes to your financial disclosure, please include your updated statement in your cover letter. Guidelines for resubmitting your figure files are available below the reviewer comments at the end of this letter.

If applicable, we recommend that you deposit your laboratory protocols in protocols.io to enhance the reproducibility of your results. Protocols.io assigns your protocol its own identifier (DOI) so that it can be cited independently in the future. For instructions see: <https://journals.plos.org/plosone/s/submission-guidelines#loc-laboratory-protocols>. Additionally, PLOS ONE offers an option for publishing peer-reviewed Lab Protocol articles, which describe protocols hosted on protocols.io. Read more information on sharing protocols at <https://plos.org/protocols?utm_medium=editorial-email&utm_source=authorletters&utm_campaign=protocols>.

We look forward to receiving your revised manuscript.

Kind regards,

Shahnawaz Anwer, PhD
Academic Editor
PLOS ONE

Journal Requirements:

1. When submitting your revision, we need you to address these additional requirements.

Please ensure that your manuscript meets PLOS ONE's style requirements, including those for file naming. The PLOS ONE style templates can be found at
<https://journals.plos.org/plosone/s/file?id=wjVg/PLOSOne_formatting_sample_main_body.pdf> and
<https://journals.plos.org/plosone/s/file?id=ba62/PLOSOne_formatting_sample_title_authors_affiliations.pdf>

2. Thank you for stating the following financial disclosure: [FK received funding from Research Fund of the National Research, Development and Innovation Office (K 147788),
AK received funding by the EKÖP-24 University Excellence Scholarship Program of The Ministry for Culture and Innovation from the Source of The National Research, Development And Innovation Fund (Grant number: EKÖP-24-4-II-ELTE-543)].

Please state what role the funders took in the study. If the funders had no role, please state: ""The funders had no role in study design, data collection and analysis, decision to publish, or preparation of the manuscript.""

If this statement is not correct you must amend it as needed.

Please include this amended Role of Funder statement in your cover letter; we will change the online submission form on your behalf.

We modified the cover letter and put in the following: The funders had no role in study design, data collection and analysis, decision to publish, or preparation of the manuscript.

3. Thank you for uploading your study's underlying data set. Unfortunately, the repository you have noted in your Data Availability statement does not qualify as an acceptable data repository according to PLOS's standards.

At this time, please upload the minimal data set necessary to replicate your study's findings to a stable, public repository (such as figshare or Dryad) and provide us with the relevant URLs, DOIs, or accession numbers that may be used to access these data. For a list of recommended repositories and additional information on PLOS standards for data deposition, please see <https://journals.plos.org/plosone/s/recommended-repositories>.

We have uploaded the data and analysis to Figshare, and also modified the doi in the manuscript (10.6084/m9.figshare.28120619)

[Note: HTML markup is below. Please do not edit.]

Reviewers' comments:

Reviewer's Responses to Questions

**Comments to the Author**

1. Is the manuscript technically sound, and do the data support the conclusions?

The manuscript must describe a technically sound piece of scientific research with data that supports the conclusions. Experiments must have been conducted rigorously, with appropriate controls, replication, and sample sizes. The conclusions must be drawn appropriately based on the data presented.

Reviewer #1: Partly

Reviewer #2: Partly

2. Has the statistical analysis been performed appropriately and rigorously?

Reviewer #1: Yes

Reviewer #2: Yes

3. Have the authors made all data underlying the findings in their manuscript fully available?

The [PLOS Data policy](http://www.plosone.org/static/policies.action#sharing) requires authors to make all data underlying the findings described in their manuscript fully available without restriction, with rare exception (please refer to the Data Availability Statement in the manuscript PDF file). The data should be provided as part of the manuscript or its supporting information, or deposited to a public repository. For example, in addition to summary statistics, the data points behind means, medians and variance measures should be available. If there are restrictions on publicly sharing data—e.g. participant privacy or use of data from a third party—those must be specified.

Reviewer #1: Yes

Reviewer #2: Yes

4. Is the manuscript presented in an intelligible fashion and written in standard English?

PLOS ONE does not copyedit accepted manuscripts, so the language in submitted articles must be clear, correct, and unambiguous. Any typographical or grammatical errors should be corrected at revision, so please note any specific errors here.

Reviewer #1: Yes

Reviewer #2: Yes

5. Review Comments to the Author

Please use the space provided to explain your answers to the questions above. You may also include additional comments for the author, including concerns about dual publication, research ethics, or publication ethics. (Please upload your review as an attachment if it exceeds 20,000 characters)

**Reviewer #1:** Article theme and research question:
The article clearly defines its theme, focusing on the impact of acute stress on individual proprioceptive accuracy (PAc). This research question holds significant importance in the fields of exercise and neuroscience.

Literature review:

The literature review section generally covers research results in related fields, but it lacks references to some of the latest or key studies, which may limit readers' comprehensive understanding of the research background. It is suggested that the author should include more recent literature directly related to this study in the revision, to enhance the timeliness and depth of the review.

Thank you for this suggestion, we added more recent literature in the light of the integrated model of the effect of anxiety on perceptual-motor performance to the introduction section. please see: “Nieuwenhuys and Oudejans [24,25] proposed an integrated model of how anxiety affects perceptual-motor performance, on the attentional, interpretational, and physical response levels. According to the model, anxiety draws attention to threat-related stimuli instead of task-relevant stimuli, leads to interpretation of ambiguous stimuli as threatening, and results in increased muscle tension and a tendency for avoidance behavior. Proprioceptive accuracy can deteriorate with allocating attention to another stimulus [26–28]. Also, an increased level of muscle tension might influence how proprioceptive stimulus is perceived [29], and the fact that extension is associated with avoidance.”

Sample Selection

The sample primarily comprises first-year college students, with a disproportionately high number of females, potentially compromising the representativeness of the sample. It is advised that the author considers a broader sample base in subsequent research to improve the study's generalizability and applicability.

Thank you for this suggestion, the authors will try to pay more attention to the representativeness of the sample in the future. We explained the considerations with the sample more broadly in the limitations and further directions section.

Data Collection and Analysis: The methods of data collection are generally reasonable, albeit with some technical challenges, such as the loss of certain physiological data (ECG and SCL), which could potentially impact the accuracy and reliability of the findings. Of course, the author can incorporate age groups and gender as subgroups in the study later on to see if different results would be obtained.

Thank you, the authors totally agree and mentions that “Technical issues that lead to the loss of physiological data (ECG and SCL) may reduce the statistical power and potentially impact the accuracy and reliability of the findings of the present study. These problems may have contributed to inconclusive findings” in the limitations section

Research Results:

The research outcomes are largely lucid, albeit with some ambiguous expressions or inadequate explanations. For instance, the elucidation of certain statistical results (e.g., non-significant outcomes) is inadequate, lacking exploration into potential causes or implications. The intriguing aspect of how to induce psychological stress and simultaneously manage physiological responses between the experimental and control groups deserves a detailed elucidation from the author.

Thank you for the feedback; we have revised the manuscript to address these concerns by providing more detailed explanations. Please see the following: “Despite the substantial increase of perceived stress, the experimental manipulation did not decrease PAc, as assessed with the active and passive versions of the Joint Position Reproduction test for the elbow joint. Thus, we could not replicate the results of Şenol and colleagues [31]. There are multiple possible explanations for this contradiction. An important difference between the two studies is that the current one used an experimental setup and a true randomized design, while the original utilized a quasi-experimental design. The lack of control group and randomization in the study of Şenol and colleagues [31] means that a third variable might have caused the reported significant association. So, the null finding can reflect the lack of effect in the population, meaning that proprioceptive accuracy does not deteriorate with acute psychological stress. From a practical point of view, this would mean that when measuring proprioceptive accuracy, for example in sports or rehabilitation context, one does not have to worry about an acute psychological stressor that would influence the results before the measurement. Also, as proprioceptive accuracy might mostly play a role in the initial (cognitive) phase of motor skill acquisition, an acute psychological stressor would not disrupt this process. A possible explanation for the null findings in the context of the integrated model of perceptual-motor performance [24], is that the effect of anxiety might be compensated with different strategies. For example, with increased mental effort, people can inhibit anxiety-related stimuli and concentrate on task-relevant stimuli. Also, people might use anxiety-reducing strategies. It is possible that participants of this study could successfully use these strategies.”

Additionally, it is pertinent to inquire whether the timing of acute stress exposure impacts the results, specifically whether there are differences in proprioceptive accuracy at 10 seconds, 30 seconds, 1 minute, and 5 minutes following acute psychological stress. It is recommended that the author conduct a detailed analysis of this matter.

Reply: Unfortunately, the PAc test needs to be administered and evaluated as a whole (i.e., consisting of 24 task), i.e., one cannot calculate individual indices for each reposition task. Thus, it is not possible run the recommended analysis. This point is discussed in the revised version. “Moreover, it is not clear how long the psychological stress induced by our stress paradigm lasts. Completing the active and passive versions of the PAc test in the study took approximately 20 minutes. Thus, it is possible that participants’ perceived stress levels substantially decreased by the end of the second PAc test. Dividing attention can decrease PAc [26]; thus, if one administers a stress task simultaneously, it won’t be clear that an eventual decrease in PAc should be interpreted as the result of the stress or the result of the decreased cognitive capacity.”

Moreover, there exists a discernible disparity between the research findings and the anticipated objectives, specifically, acute stress did not significantly impact proprioceptive accuracy. The author ought to undertake a more comprehensive analysis and discussion to pinpoint research limitations and future research trajectories.

Thank you very much for the suggestion, we dedicated the “limitations and future directions” section to this issue in the revised manuscript.

Discussion and Conclusion

The discussion section primarily covers the significance and limitations of the research findings, yet it lacks an in-depth exploration of certain key issues. For instance, the author fails to adequately explain why acute stress did not affect proprioceptive accuracy, and how this finding relates to existing literature.

Thank you for your suggestion, we expanded the section where we explain these results. please see: “An important difference between the two studies is that the current one used an experimental setup and a true randomized design, while the original utilized a quasi-experimental design. The lack of control group and randomization in the study of Şenol and colleagues [31] means that a third variable might have caused the reported significant association.” and “As a substantial difference in perceived stress level was found, the lack of difference with respect to the physiological indicators of the stress response can be explained by the well-known dissociation between bodily changes and their perceived counterparts (for a review, see [44]).” and “A further option is that the decrement in PAc is related to the physiological aspect of the stress response, not to the perceived aspect. For example, it is possible that increased internal noise due sympathetic activation has a negative impact on the low-level processing of the proprioceptive signal.” and “Another important point to discuss is that when evaluating the results related to proprioception, it is always important to consider the joint measured, and the method used [2]. For this study, we used the Joint Position Reproduction test with active and passive versions for the right elbow joint, while Şenol and colleagues [31] used the Joint Position Reproduction test for the ankle joint only with active reproduction. It cannot be excluded that stress influences the accuracy shown in different body sites differently.”

The conclusion section is relatively concise, yet it lacks clear recommendations or directions for future research. It is suggested that the author add more prospects and suggestions for future research in the conclusion section to guide further development in this field. In summary, this article possesses certain theoretical and practical significance in exploring the impact of acute stress on proprioceptive accuracy.

Thank you, the conclusion section is expanded with some recommendations for future research. “Future research could focus not only acute but on chronic stress, or could use multiple stress-induction methods, and more proprioceptive tasks to better understand the relationship between stress and proprioceptive accuracy.”

The overall language of the article is fluent, and the chart production is generally standardized. However, there are still some shortcomings in research methods, sample selection, data collection and analysis, interpretation of research results, and discussion and conclusions. It is recommended that the author can consider the aforementioned suggestions during revision.

Thank you for these suggestions. We have created a new “Limitations and future directions” part, where we reflect on the limitations, and based on these, we give clear recommendations for future research directions.

**Reviewer #2:** Thanks for receiving the opportunity to review your paper. Overall, I appreciate the thoroughness and importance of your study on acute psychological stress does not influence joint position reproduction performance in the elbow joint. However, this manuscript must undergo minor revisions before it can be considered for publication.

Dear Reviewer, Thank you for your comments. The authors will address all recommendations. Please find our responses below.

1. The abstract is well-structured but could benefit from a more concise summary of key results. the keyword should in MeSH terms. The results section should reflect the statistical values of the outcomes.

The results section of the abstract has been made more concise.

2. The introduction is comprehensive but could be streamlined to avoid redundancy, particularly in the explanation of stress mechanisms and proprioception.

The mentioned sections were streamlined.

3. The double-blind design and randomization are strong aspects of the methodology. However, the limitations of using a homogeneous sample (primarily young university students) should be emphasized more prominently in the discussion.

We added more emphasis to this in the limitations and future directions section. Please see the following part: “An important limitation of the current study is that the sample was homogeneous as the study involved first year university students, 86% of whom were female. This limits the generalizability of the findings. For future studies, it is recommended to use a more diverse, preferably representative sample. It is possible that the stress effect on proprioceptive accuracy is not linear and shows high variability across individuals. Future studies should also test these possibilities.”

4. The reliance on self-reported stress data and the loss of physiological data due to technical issues (e.g., ECG and SCL) weaken the reliability of the findings. Future studies should address these issues to strengthen conclusions.

Thank you for your suggestion, we will pay more attention to these problems and to avoid data loss in the future. The issue is also highlighted in the limitations section now.

5. The results indicate inconclusive findings for some measures. Adding a discussion on why these results were inconclusive (e.g., sample size, experimental design) would enhance the reader's understanding.

Thank you for this comment, the authors mention in the limitation section that the possibility of limited statistical power due to sample size, technical issues leading to missing physiological data, and the stressor's limited ecological validity could contribute inconclusive findings. We added the following sentence to the limitation secion: “These problems may have contributed to inconclusive findings.”

6. The discussion effectively highlights the discrepancy between perceived stress and physiological responses. However, further exploration of how these results fit within existing literature (beyond Şenol et al.) would provide more depth. The lack of effect on proprioceptive accuracy is explained well, but implications for broader contexts (e.g., sports, rehabilitation) could be expanded.

Thank you, the discussion section is expanded based on this comment.

7. The tables are clear, but the presentation of descriptive statistics (e.g., in Table 1) could be visually improved with better formatting.

Thank you, the table is slightly reformatted.

8. Figures are informative, though labels and legends could be enhanced for clarity

Thank you, we have tried to make the figures clearer.

9. While limitations are acknowledged, they could be more critically analyzed, particularly regarding ecological validity and the generalizability of findings.

Thank you, thank you, now we put more emphasis on these limitations in the first and second paragraph of the limitations section. Please see: “An important limitation of the current study is that the sample was homogeneous as the study involved first year university students, 86% of whom were female. This limits the generalizability of the findings. For future studies, it is recommended to use a more diverse, preferably representative sample. It is possible that the stress effect on proprioceptive accuracy is not linear and shows high variability across individuals. Future studies should also test these possibilities.

Also, the ecological validity of the quiz-based stressor used in this study is limited as it represents a test or exam like acute stress which may have smaller or different effects than a real-life stressor, not to speak of longer-term chronic stress.”

10. Future research directions should include testing a more diverse population and incorporating chronic stress models.

Thank you for this comment, despite the difficulties in recruiting a more diverse population, we will try to move in this direction in the future. We have included this consideration in the limitations section.

6. PLOS authors have the option to publish the peer review history of their article ([what does this mean?](https://journals.plos.org/plosone/s/editorial-and-peer-review-process#loc-peer-review-history)). If published, this will include your full peer review and any attached files.

If you choose “no”, your identity will remain anonymous but your review may still be made public.

**Do you want your identity to be public for this peer review?** For information about this choice, including consent withdrawal, please see our [Privacy Policy](https://www.plos.org/privacy-policy).

Reviewer #1: No

Reviewer #2: No
